# A meta‐analytic review of attitudes towards the sexuality of adults with intellectual disabilities as measured by the ASQ‐ID and related variables: Is context the key?

**DOI:** 10.1111/jir.12971

**Published:** 2022-08-15

**Authors:** A. B. Correa, J. D. Moreno, A. Castro

**Affiliations:** ^1^ Department of Psychology and Sociology, Faculty of Social and Human Sciences University of Zaragoza Teruel Spain; ^2^ Departament of Basic Psychology, Faculty of Psychology Autonomous University of Madrid Madrid Spain

**Keywords:** ASQ‐ID, Attitudes, Intellectual disability, Meta‐analysis, Sexuality

## Abstract

**Background:**

The attitudes of others towards the sexuality of people with intellectual disabilities are one of the main perceived barriers to them expressing their sexuality. Research on what influences these attitudes yields heterogeneous results.

**Method:**

A systematic review of the literature and a meta‐analysis were carried out.

**Results:**

Eleven studies using the Attitudes to Sexuality Questionnaire—Individuals with an Intellectual Disability (ASQ‐ID) were included. Within the included studies, the country's socio‐economic development and level of individualism were associated with attitudes towards the sexual rights, parenting and self‐control of adults with intellectual disabilities. General population and staff samples held more favourable attitudes than family samples in terms of sexual rights and parenting. Age and gender did not yield significant results.

**Conclusions:**

Variables related to country context may underlie the differences observed between countries and therefore influence the population's general thinking and ideologies. Unexpectedly, no age differences were observed. Gender‐related results may reflect rapprochement between genders in sexuality. These findings are relevant for researchers and practitioners, as they suggest the importance of considering contextual factors when developing effective interventions that aim to support adults with disabilities to live their sexuality.

## Introduction

Living and expressing our sexuality is a right. However, in practice, not everyone enjoys this right. The sexuality of adults with intellectual disabilities (ID) is surrounded by myths and stigma (Pebdani and Tashjian 2022), which directly interfere with their experience of sexuality. The perceptions of others are one of the main barriers to the sexuality of adults with ID (Sinclair *et al*. 2015), and these attitudes may be key to explaining why sexuality issues are not adequately reflected in individual support plans (Stoffelen *et al*. 2017). Adults with ID themselves report how influential the attitudes of staff carers and family members are on their experience of sexuality, which sometimes needs to be experienced with secrecy and deception (Healy *et al*. 2009).

The attitudinal landscape appears to be changing with regard to these perceptions. Prior to the 2000s, the sexuality of adults with ID was not at the forefront of researchers' agendas (Aunos and Feldman 2002), and attitudes were not entirely favourable. In the last two decades, these attitudes seem to have somewhat improved (Correa *et al*. 2022), although they are not as inclusive as they should be. If we intend to intervene on these attitudes, with the aim of improving the support given to adults with ID to express their sexuality as they wish, we should carefully analyse the current attitudinal scenario. More specifically, we should try to address what these attitudes are like and what factors may be influencing less favourable attitudes or misconceptions. Different variables have been proposed to explain what would influence more or less favourable attitudes, with different results across studies. The type of relationship maintained with disabilities, cultural aspects, age and gender have been singled out as being relevant to this issue (Correa *et al*. 2022).

Regarding the type of relationship, people with a family member with ID, staff carers for people with ID and general population samples (not directly related to someone with ID) hold different attitudes. Apparently, general population samples seem to hold more favourable attitudes, followed by sector workers and family members (Aunos and Feldman 2002; Lam *et al*. 2021; Correa *et al*. 2022).

Attitudes towards the sexuality of people with ID have not been found to be equally favourable in all countries. A comparison between five different countries with a common measure revealed apparent differences (no statistical analysis was performed) between them, finding that White Westerns in the UK held the most favourable attitudes and Indonesian participants the least (Winarni *et al*. 2018). Moreover, people from different backgrounds showed different attitudes even when living in the same country. South Asians living in the UK had less favourable attitudes than White Westerns living in the UK (Sankhla and Theodore 2015). Although this study proposes that acculturation might be occurring, and that South Asians living in the UK may be more favourable than those living abroad, differences emerge.

Previous studies have explored differences according to cultural orientation variables. One study found that up to 27% of the variability in attitudes may be explained when considering measures of individualism and collectivism (Ditchman *et al*. 2017). According to the latter study, the constructs of individualism or collectivism are a fundamental way of examining culture. Societies that are more individualistic or collectivistic differ from each other in significant cultural aspects, such as social norms and values (Hofstede *et al*. 2010), both of which are directly associated with how people relate to each other and how sexuality is addressed in a geographical area. In fact, individualism has already been related to general sexuality and has even shown a positive correlation with behavioural aspects such as sexual frequency (Ubillos *et al*. 2000). However, it should not be ignored that the degree of individualism of a country is often related to a higher socio‐economic development (Hofstede *et al*. 2010). In addition, the socio‐economic development of a country may be related to the acceptance of self‐expressive sexual values, among others (Lottes and Alkula 2011), so a similar pattern in attitudes towards the sexuality of adults with ID could be expected.

Age has been extensively examined in relation to attitudes towards the sexuality of people with ID (Lam *et al*. 2021; Correa *et al*. 2022; Pebdani and Tashjian 2022). Older age appears to be associated with less favourable attitudes (Karellou 2003; Cuskelly and Bryde 2004; Esterle *et al*. 2008), reflecting the ideological paradigms of each generation in terms of sexuality. Although some studies did not replicate these findings (Ryan and McConkey 2000; Sankhla and Theodore 2015), this difference has been proposed as an explanatory hypothesis for differences between samples (Cuskelly and Bryde 2004; Evans *et al*. 2009).

Finally, gender has also been proposed as a moderator variable, yielding heterogeneous results (Lam *et al*. 2021; Correa *et al*. 2022). Some studies found that women have more favourable attitudes than men in some aspects such as their right to an affective or sexual life, marriage or sex education (Franco *et al*. 2012; Ditchman *et al*. 2017), whereas others did not replicate this relationship. One study found that men had more favourable attitudes towards parenting and sex education among adults with ID (Ryan and McConkey 2000). Another study found that women had less favourable attitudes than men regarding socio‐sexual behaviours among people with ID in the context of different‐gender relationships, but more favourable than men in the context of same‐gender relationships (Oliver *et al*. 2002). Other studies found no relationship between respondents' gender and attitudes (Karellou 2003; Cuskelly and Bryde 2004; Grieve *et al*. 2009).

One difficulty in comparing results across studies is the heterogeneity of the approaches used (Morales *et al*. 2010). According to previous review studies, the Attitudes to Sexuality Questionnaire—Individuals with an Intellectual Disability (ASQ‐ID) was found to be the most frequently used measure in different projects and countries (Lam *et al*. 2021; Correa *et al*. 2022). Up to eight studies using the ASQ‐ID measure in its final form were identified, followed by a case‐scenario proposal used in three studies, the POS and GSAQ‐LD scales used in two studies, and the SAQ and SMRAI measures, used in only one study each (Correa *et al*. 2022). Other studies used self‐generated interviews and questionnaires to assess attitudes, the use of which was not widespread to other studies as much as the ASQ‐ID (Correa *et al*. 2022). The ASQ‐ID (Cuskelly and Gilmore 2007) is a 28‐item scale that covers different aspects of sexuality, such as those related to the sexual rights of adults with ID, the capacity or possibility of the adult with ID to be a parent, the right to maintain non‐reproductive sexual behaviours and the capacity for self‐control (related to sexuality).

The current attitudinal paradigm has recently been systematically reviewed, with interesting but heterogeneous results (Lam *et al*. 2021; Correa *et al*. 2022; Pebdani and Tashjian 2022). However, to our knowledge, no study has attempted to compile data from different studies with a statistical approach to try to clarify this issue. Therefore, the present study aims to examine how attitudes are influenced by previously reported variables in different studies and countries using meta‐analytic methods. Given that the ASQ‐ID has been identified as the most commonly used measure, four independent meta‐analyses were conducted for the different ASQ‐ID subscales, including all the identified primary studies using this measure. In addition, the potential moderation role of several variables of interest were tested, such as the sample type (family, staff or general population), variables related to country of origin (individualism and socio‐economic development), age and gender.

## Method

The present research conducted a meta‐analytic review of studies on attitudes towards the sexuality of adults with intellectual disabilities, as assessed by the ASQ‐ID questionnaire. This meta‐analytic review was conducted according to the Preferred Reporting Items for Systematic Reviews and Meta‐Analysis (PRISMA‐P) tool (Moher *et al*. 2015). The corresponding review protocol was registered on the open science framework network (https://osf.io/b9f37) and the PRISMA 2020 checklist is available as Data S1.

This meta‐analytical review is part of a broader project in the field of attitudes towards the sexuality of adults with intellectual disabilities, which began with a general and broad systematic review by Correa *et al*. (2022). After this first approach, it was observed that several studies used the ASQ‐ID measure. This made it possible to carry out a statistical approach to the issue, which had not been previously attempted and had two main purposes. (1) Try to confirm the consistency of some of the previously reported outcomes across studies, with a statistical support, giving strength to any conclusions made. (2) To draw out broader data related to the context of different countries with regard to attitudes, such as individualism and GDP per capita, and to perform a comparison that had not previously been possible, supported by statistical analysis which could evaluate different studies. Compared with that first broad approach, this meta‐analysis offers more specific and strong conclusions and allows us to explore new contextual and cultural factors that were not adequately explored in our previous work nor previous literature.

### Search strategy and selection of studies

**Figure 1:**
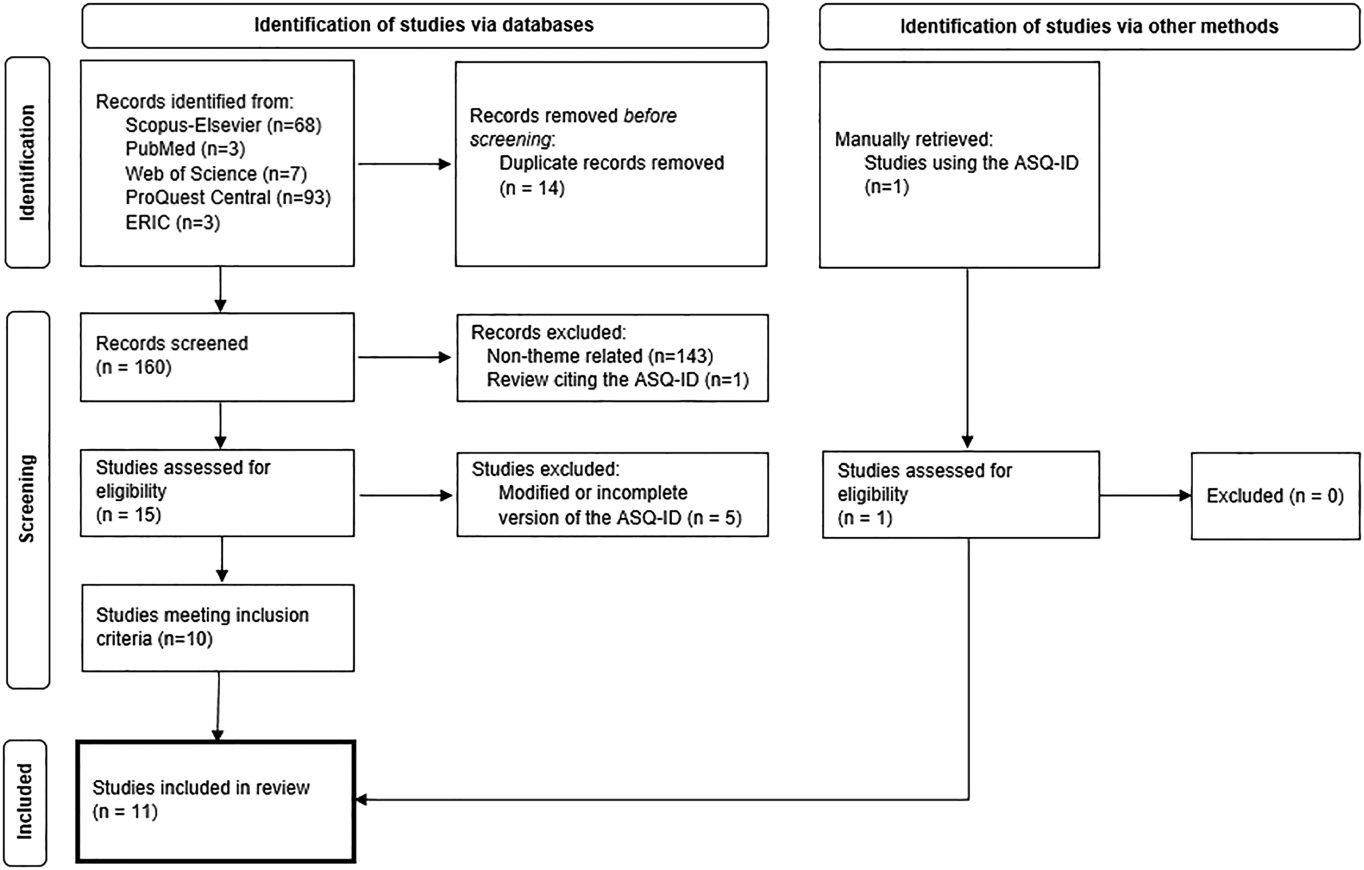
Systematic review flowchart.

Initial search and reference retrieval was conducted in July 2021 through the following databases: PubMed, Scopus‐Elsevier, ProQuest Central, Education Resource Information Centre (ERIC) and Web of Science. Search terms were introduced in Boolean search as follows: (((attitudes AND sexuality) AND (((intellectual OR learning) AND disability) OR (mental AND retardation)) AND ASQ) OR ASQ‐ID). Search results were limited to those published between September 2007 (publication date of the ASQ‐ID measure) and July 2021. The initial retrieval of references was carried out by one author, whereas the process of screening and selection for inclusion was performed independently by the first and third authors of the manuscript. There were no disagreements. This process is summarised in Fig. 1, and the descriptive statistics of the final sample of studies are shown in Table 1.

**Table 1:** Effect size estimates and 95% CI of the studies included in the meta‐analysis for each ASQ subscale

Studies	*N*	Sexual Rights	Parenting	Non‐reproductive Sexual Behaviour	Self‐Control
Chou *et al*. (2018). S1	130	3.48 [3.38;3.59]	2.62 [2.45;2.79]	4.24 [4.11;4.37]	3.50 [3.32;3.68]
Chou *et al*. (2018). S2	173	4.06 [3.97;4.15]	3.24 [3.06;3.41]	4.99 [4.88;5.10]	3.77 [3.62;3.91]
Chou *et al*. (2018). S3	645	4.34 [4.30;4.38]	4.40 [4.33;4.47]	4.99 [4.93;5.05]	4.28 [4.22;4.35]
Cuskelly and Gilmore (2007)	261	4.53 [4.43;4.63]	4.37 [4.25;4.49]	3.00 [2.91;3.09]	4.50 [4.40;4.61]
Deffew *et al*. (2022)	86	4.71 [4.58;4.84]	4.84 [4.66;5.02]	5.32 [5.17;5.47]	5.13 [4.96;5.31]
Di Marco *et al*. (2013)	122	4.34 [4.24;4.44]	4.22 [4.05;4.39]	4.53 [4.38;4.68]	4.46 [4.30;4.62]
Ditchman *et al*. (2017)	227	4.42 [4.33;4.51]	4.50 [4.38;4.62]	4.13 [4.05;4.21]	4.30 [4.18;4.42]
Gilmore and Chambers (2010). S1	169	4.81 [4.72;4.89]	4.51 [4.39;4.64]	4.90 [4.80;5.01]	4.89 [4.77;5.01]
Gilmore and Chambers (2010). S2	50	4.70 [4.55;4.85]	4.81 [4.61;5.01]	4.99 [4.85;5.12]	4.72 [4.55;4.89]
Meaney‐Tavares and Gavidia‐Payne (2012)	66	4.98 [4.84;5.11]	4.85 [4.65;5.06]	5.05 [4.85;5.24]	5.07 [4.87;5.26]
Pebdani (2016)	71	5.09 [4.96;5.23]	4.94 [4.78;5.10]	5.18 [5.02;5.34]	5.37 [5.23;5.51]
Sankhla and Theodore (2015). S1	183	4.98 [4.89;5.07]	5.11 [5.01;5.21]	5.08 [4.97;5.19]	4.92 [4.80;5.04]
Sankhla and Theodore (2015). S2	143	4.45 [4.33;4.57]	4.48 [4.31;4.65]	4.70 [4.55;4.85]	4.40 [4.20;4.60]
Tamas *et al*. (2019). S1	137	3.41 [3.32;3.49]	3.16 [3.02;3.30]	3.66 [3.55;3.76]	3.48 [3.34;3.61]
Tamas *et al*. (2019). S2	137	3.96 [3.86;4.06]	3.76 [3.60;3.92]	4.44 [4.32;4.55]	3.69 [3.54;3.84]
Tamas *et al*. (2019). S3	137	3.50 [3.41;3.58]	3.17 [3.07;3.27]	3.71 [3.65;3.76]	3.23 [3.11;3.34]
Winarni *et al*. (2018)	28	3.70 [3.62;3.78]	3.50 [3.39;3.61]	3.40 [3.23;3.57]	3.40 [3.13;3.67]

N, sample size; S1, Study 1; S2, Study 2; S3, Study 3.

The four columns to the right show the effect size and its 95% CI calculated for each measure obtained from each of the studies included in the meta‐analysis.

### Inclusion criteria

Studies included in this review had to meet the following inclusion criteria: (1) used the ASQ‐ID measure (Cuskelly and Gilmore 2007); (2) used the original, full version of the ASQ‐ID, not a modified or partially applied one; and (3) included or provided data on at least one of the following characteristics of the sample: country of origin, mean age, percentage of women or specify the type of sample responding to the scale (family, staff or general population).

### Data extraction and treatment

The three study authors agreed and designed a CSV data extraction sheet to extract and process the data from the included studies. This sheet was completed by one author and reviewed by a second author, with no disagreements arising. For those studies that included information from different subsamples (family, staff or general population), each subsample was recorded independently. The data collected on this sheet included the mean response scores (and standard deviation) for each subscale of the ASQ‐ID and data for the six selected moderator variables (commented in the moderator variables section). This sheet was also used to conduct the necessary data transformations.

### ASQ measurement and data transformations

The selected studies provided a full administration of the original ASQ‐ID measure (Cuskelly and Gilmore 2007). The ASQ‐ID is a 28 items (in its final version) scale, which aims to assess general attitudes towards the sexuality of adults with a mild to moderate intellectual disability. This scale is answered on a 6‐point Likert scale, where 1 = *strongly disagree* and 6 = *strongly agree*. Rather than using an overall mean score, ASQ‐ID scores are typically reported for its four subscales, which are attitudes towards sexual rights (13 items), parenting (seven items), non‐reproductive sexual behaviour (five items) and self‐control (three items). However, when collecting data from the included studies, not all of them provided the data in the same way so some transformations had to be made.

First, some studies conducted a dual‐gendered administration of the ASQ‐ID. Thus, the same questions were administered in two sets of questionnaires, versioned for women and men with ID (a common practice when administering the ASQ‐ID). In order to compare data across studies, a single non‐gendered measure was calculated for each subscale. For each subscale, we calculated weighted means and standard deviations of the scores for men and women with ID.

Second, two studies (Pebdani 2016; Deffew *et al*. 2022) scored the ASQ‐ID from 0 to 5, instead of scoring from 1 to 6. Therefore, the corresponding constant (13, 7, 5 or 3) was added to the mean score of each subscale (overall mean scores were provided). Once transformed, the overall mean scores needed a final transformation (described below).

Third, some studies gave an overall subscale score (Cuskelly and Gilmore 2007; Gilmore and Chambers 2010; Pebdani 2016; Chou *et al*. 2018; Deffew *et al*. 2022), rather than a mean response score for each subscale (used value, allowing for intra‐study comparisons between subscales and not just across studies). Therefore, these overall means and standard deviations were converted according to the ASQ‐ID structure data (dividing by 13, 7, 5 or 3) to obtain mean response scores for each subscale.

Fourth, and related to moderator variables, some studies did not report a mean value for age (Cuskelly and Gilmore 2007; Gilmore and Chambers 2010; Meaney‐Tavares and Gavidia‐Payne 2012; Sankhla and Theodore 2015; Winarni *et al*. 2018; Deffew *et al*. 2022). In these cases, the study authors were contacted in an attempt to obtain these data. The same was done for gender in the study of Winarni *et al*. (2018), who provided the corresponding data. For those cases where a response on mean age was received, these data were reported as not available due to the method of data collection (by age ranges). For these studies, a mean age value was calculated by generating the weighted mean value of the means of the age ranges provided.

### Quality assessment

All the studies that met the inclusion criteria were included for analysis, without quality assessment being an exclusion or inclusion criteria. However, with the exception of one study (Di Marco *et al*. 2013), all the included studies were published in peer‐reviewed academic journals, ensuring minimum quality standards.

### Effect size calculation and statistical analysis

Given the design of the studies analysed, the effect size measure selected for the present meta‐analysis was the sample mean. The heterogeneity of the estimations was analysed by *Q* tests and *I* ^*2*^ indexes (Huedo‐Medina *et al*. 2006). Statistical analyses assumed a random‐effects model, as it is more conservative than a fixed‐effects model, allowing generalising conclusions beyond the specific set of studies analysed (Hedges and Vevea 1998; Raundenbush 2009; Borenstein *et al*. 2010). The combined estimates were calculated weighting the individual studies by the inverse of their variances. The method used to estimate between‐study variability was the Hartung–Knapp–Sidik–Jonkman method for random‐effects meta‐analysis (IntHout *et al*. 2014). All the statistical analyses were performed with R Statistical Software (R Core Team 2021) in the 4.1.2 version, using the *metafor* package—Version 3.0‐2 (Viechtbauer 2010a, 2010b) for the combined estimates, the *Q* statistic, the *I* ^*2*^ statistic estimates and the meta‐regression analyses for the continuous moderator variables. We also used the *meta* package to generate the forest plots—Version 5.1‐1 (Schwarzer 2007). In addition, we used the SPSS macros of Lipsey and Wilson (2001) to analyse the categorical moderator variables. To test the publication bias, we calculated Kendall's tau and the Egger's test (Borenstein *et al*. 2010; Botella and Sánchez‐Meca 2015), also through *metafor*. Separate meta‐analyses were performed for each of the four subscales.

### Moderator variables

In order to analyse the heterogeneity between the results of the studies, we conducted several moderator analyses for each of the four subscales. Six different moderator variables were selected based on their potential explanatory role in the results of the analysed studies. On the one hand, four categorical moderator variables were included in the analyses. First, to assess the socio‐economic development of the samples, we included data on the annual per capita income level of the participants' country of origin, measured by the gross domestic product (GDP) per capita in dollars (<25 000$; >25 000$), as a moderator variable. Data from the human development reports data centre (available at: https://hdr.undp.org/en/indicators/194906#) in 2018 were used. Data for Taiwan was not available, so based on previous studies (Bergmüller 2013), data from the world factbook (Central Intelligence Agency n.d.) were used instead. It was possible to retrieve GDP data in 2018. Second, we considered the different country origins of the participants (classified by continents, North America, Oceania, Asia and Europe) as a moderator variable. Third, we also included the type of participant sample from the primary studies (family, staff and general population) as another moderator variable. Finally, we considered the level of individualism attributed to each country as a moderator variable. The level of individualism–collectivism of a significant number of countries has previously been assessed by IBM studies in the form of an individualism index, as reported by Hofstede *et al*. (2010). This index is reported with scores from 0 to 100, in which higher ratings refer to more individualistic countries and lower scores to more collectivistic countries. For this meta‐analysis, the individualism scores reported by Hofstede *et al*. (2010) for the countries of the included studies were retrieved and considered in a dichotomous format (<50; >50) for the statistical analysis. Furthermore, two continuous moderator variables were also included in the analyses, the mean age of the participants of the primary studies and the gender (in proportions) of these participants.

## Results

A total of 11 studies using the ASQ‐ID measure were retrieved. Studies including family, staff and/or general population samples were carried out in the UK (Sankhla and Theodore 2015), Ireland (Deffew *et al*. 2022), the USA (Pebdani 2016; Ditchman *et al*. 2017), Australia (Cuskelly and Gilmore 2007; Gilmore and Chambers 2010; Meaney‐Tavares and Gavidia‐Payne 2012), Italy (Di Marco *et al*. 2013), Taiwan (Chou *et al*. 2018), Serbia (Tamas *et al*. 2019) and Indonesia (Winarni *et al*. 2018).

### Combined effect size estimates

**Figure 2:**
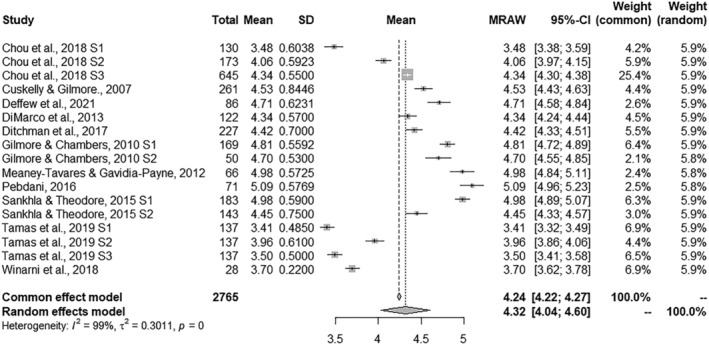
Forest plot for Sexual Rights.

**Figure 3:**
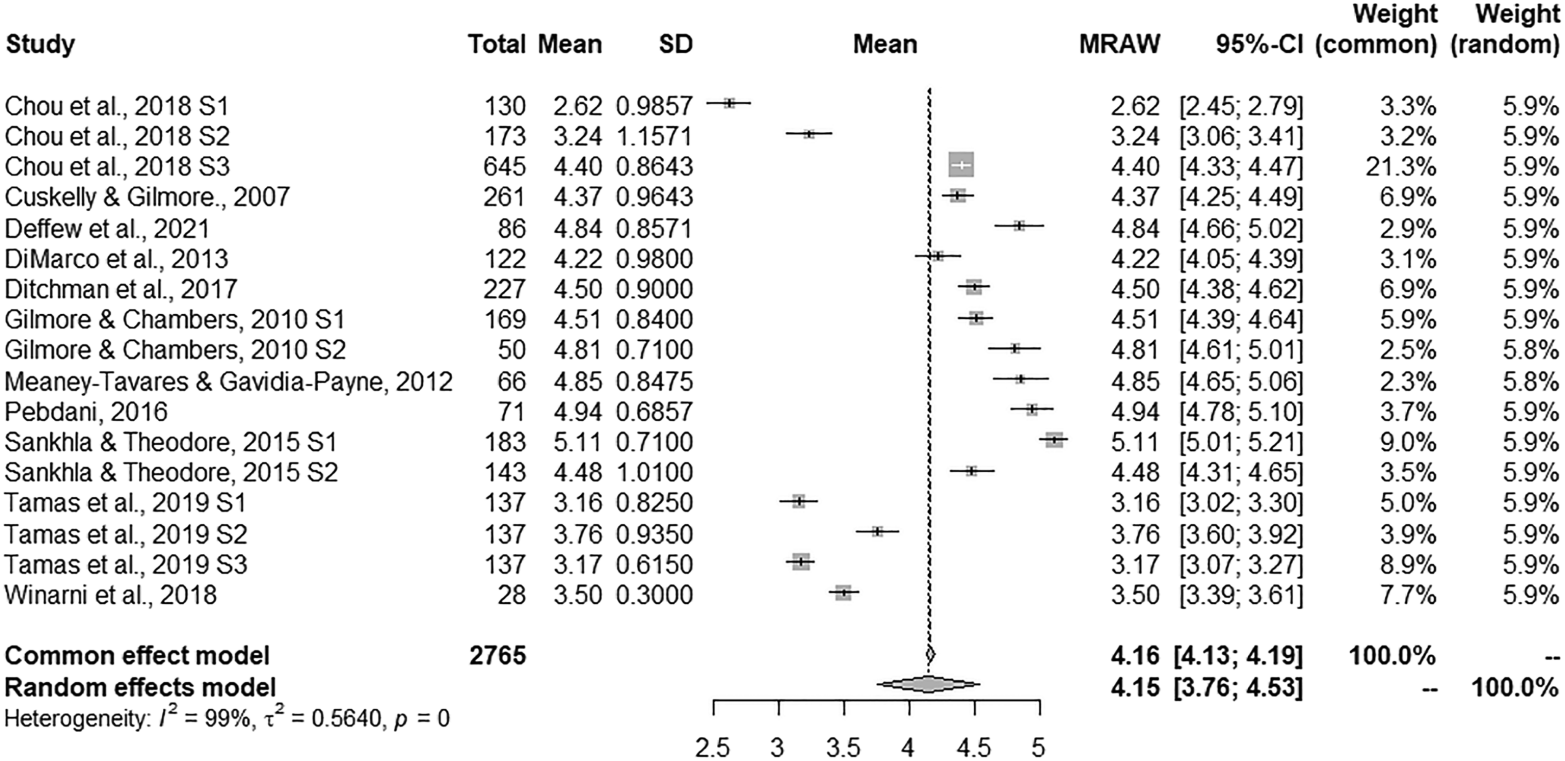
Forest plot for Parenting.

**Figure 4:**
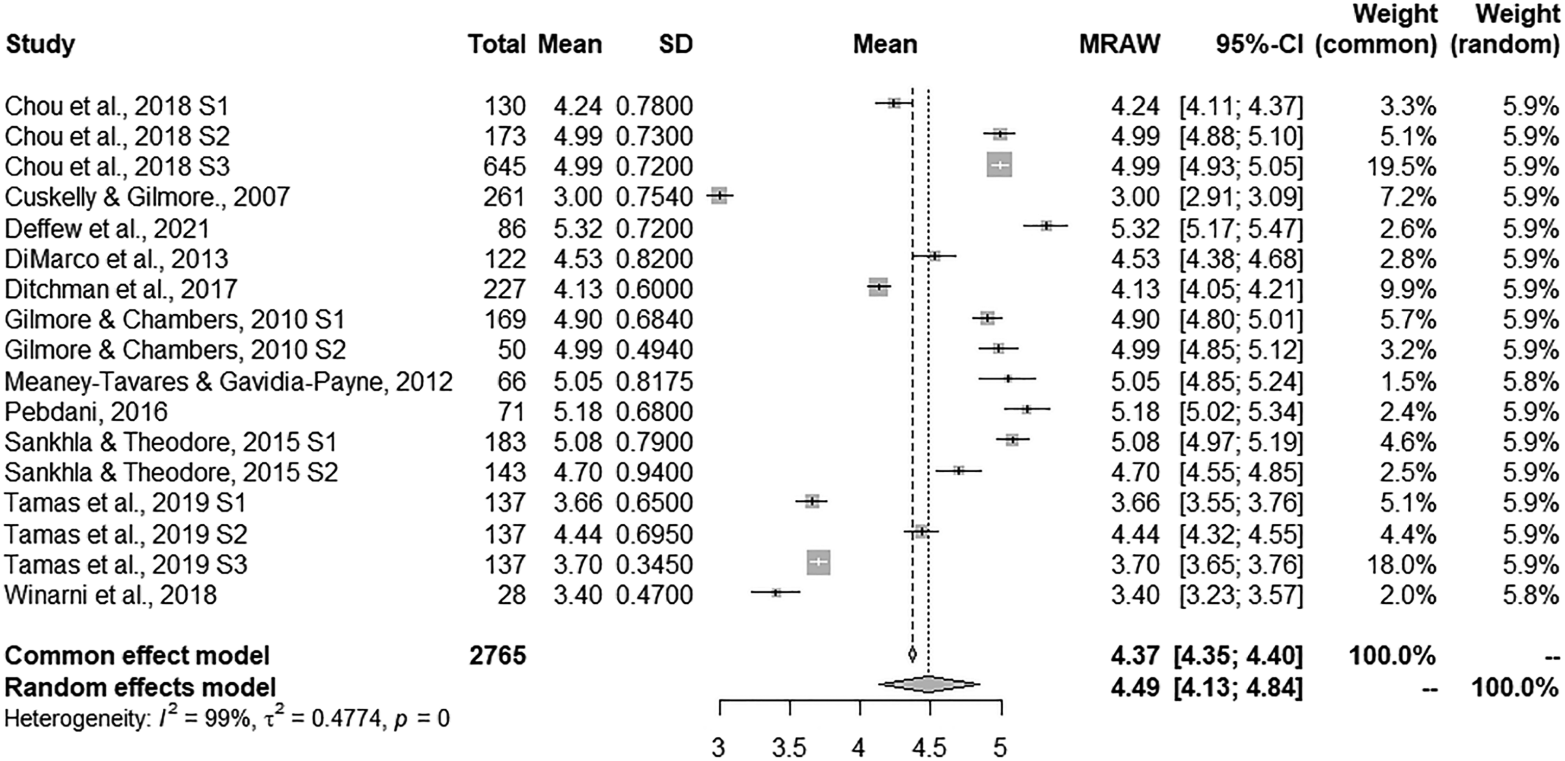
Forest plot for Non‐reproductive Sexual Behaviour.

**Figure 5:**
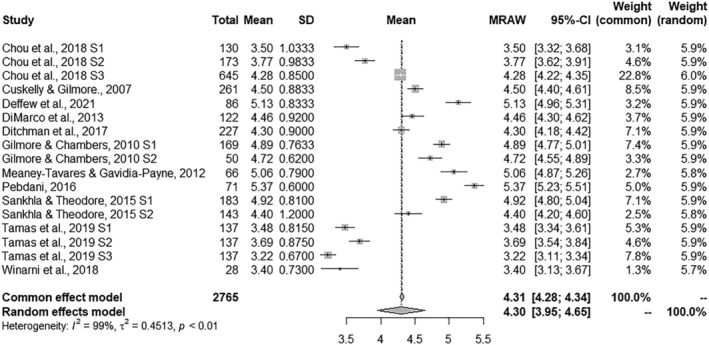
Forest plot for Self‐Control.

A summary of the results obtained for the four ASQ subscales is shown in Table 2. The combined effect size estimates offer information about the general mean for the set of studies in each of the subscales. The combined effect sizes ranged from 4.15 to 4.49 for each dependent measure. Significance tests were performed with the Hartung–Knapp–Sidik–Jonkman method (IntHout *et al*. 2014). The effect size was positive for the four measures and all of them reached *p*‐values under .001. The forest plots presented in Figs 2, 3, 4, 5 provide a graphical overview of the effects studied for the four ASQ subscales. In addition, all homogeneity tests showed significant values for the *Q* statistic (*P* < 0.001), allowing the null hypothesis of homogeneity for the four measures to be rejected. In accordance, the values of the *I* ^*2*^ statistic reached high values, ranging from 99.01 to 99.42%, indicating that the heterogeneity was considerably higher than expected from random sampling. Following the criteria proposed by Higgins and Green (2011), the heterogeneity of the four measures should be assessed as considerable (>75%). Thus, for all the measures, there was a large amount of heterogeneity that could be explained by the presence of potential moderator variables.

**Table 2:** Combined estimates for each ASQ subscale using a random‐effects model (significance tests with the Hartung–Knapp–Sidik–Jonkman method; IntHout *et al.* 2014)

ASQ subscale	*k*	*M*	95% CI	*t*	*Q* (df)	*I* ^2^	*τ* ^2^
Sexual Rights	17	4.32	4.04; 4.60	32.30 (*P* < 0.001)	2019.79(16) (*P* < 0.001)	99.30%	.30
Parenting	17	4.15	3.76; 4.53	22.64 (*P* < 0.001)	1844.08(16) (*P* < 0.001)	99.23%	.56
Non‐reproductive Sexual Behaviour	17	4.49	4.13; 4.84	26.66 (*P* < 0.001)	2945.65(16) (*P* < 0.001)	99.42%	.48
Self‐Control	17	4.30	3.95; 4.65	26.19 (*P* < 0.001)	1352.93(16) (*P* < 0.001)	99.01%	.45

*k* = number of studies analysed for each subscale; *M* = mean effect size (sample mean).

### Publication bias

As can be checked in Table 3, the results for Kendall's tau and Egger's test did not show any tendency of publication bias in the sample of studies analysed in the present meta‐analysis.

**Table 3:** Statistical tests for publication bias for each ASQ subscale

ASQ Subscale	Kendall's tau	Egger's test
Sexual Rights	0.29 (*P* = 0.11)	1.98 (*P* = 0.07)
Parenting	0.06 (*P* = 0.78)	0.35 (*P* = 0.73)
Non‐reproductive Sexual Behaviour	0.09 (*P* = 0.66)	1.09 (*P* = 0.29)
Self‐Control	0.04 (*P* = 0.84)	−0.49 (*P* = 0.63)

### Moderator analyses

The moderator analyses for categorical variables are summarised in Table 4. For the socio‐economic development variable according to GDP per capita (<25 000$; >25 000$), a statistically significant effect was observed for the Sexual Rights, Parenting and Self‐Control subscales. This effect was led by higher subscale scores for participants with an income level over 25 000$. According to the participant's continent of origin variable (North America, Oceania, Asia, Europe), no statistically significant differences were observed in any subscale. Regarding the type of participant sample variable (family, sector workers, general population), a statistically significant effect was observed for the Sexual Rights and Parenting subscales. This effect was led by higher subscale scores for sector workers and general population than for family samples. Finally, according to the categorization based on the individualism scores variable (<50; >50), a statistically significant effect was observed for the Sexual Rights, Parenting and Self‐Control subscales. This effect was led by higher subscale scores for participants with individualism scores over 50.

**Table 4:** Moderator analysis for categorical variables

Variable	Level	Sexual Rights				Parenting				Non‐reproductive Sexual Behaviour				Self‐Control			
		*k*	*M*	95%CI	*Q*_B_	*k*	*M*	95%CI	*Q*_B_	*k*	*M*	95%CI	*Q*_B_	*k*	*M*	95%CI	*Q*_B_
Rent	<25 000$	7	3.79	3.56; 4.02	35.64 (*P* < 0.001)	7	3.41	3.09; 3.73	35.31 (*P* < 0.001)	7	4.21	3.71; 4.71	2.04 (*P* = 0.15)	7	3.63	3.67; 3.89	42.43 (*P* < 0.001)
	>25 000$	10	4.69	4.50; 4.89		10	4.66	4.40; 4.93		10	4.68	4.27; 5.10		10	4.77	4.55; 4.99	
Culture	North America	2	4.75	4.08; 5.42	7.39 (*P* = 0.06)	2	4.72	3.76; 5.68	6.02 (*P* = 0.11)	2	4.65	3.60; 5.71	0.11 (*P* = 0.99)	2	4.83	3.99; 5.66	7.02 (*P* = 0.07)
	Oceania	4	4.75	4.27; 5.23		4	4.63	3.95; 5.31		4	4.48	3.73; 5.22		4	4.79	4.20; 5.38	
	Asia	5	4.02	3.59; 4.44		5	3.65	3.04; 4.26		5	4.48	3.81; 5.14		5	3.88	3.35; 4.41	
	Europe	6	4.15	3.76; 4.54		6	4.04	3.49; 4.60		6	4.45	3.84; 5.06		6	4.15	3.67; 4.63	
Type of sample	Family	2	3.44	2.84; 4.05	9.98 (*P* = 0.008)	2	2.89	2.06; 3.72	11.04 (*P* = 0.007)	2	3.95	3.08; 4.82	3.34 (*P* = 0.09)	2	3.49	2.66; 4.31	5.07 (*P* = 0.06)
	Sector workers	6	4.52	4.16; 4.87		6	4.25	3.77; 4.73		6	4.86	4.35; 5.36		6	4.57	4.09; 5.05	
	General population	8	4.46	4.16; 4.77		8	4.46	4.04; 4.87		8	4.48	4.04; 4.91		8	4.41	4.00; 4.82	
Individualism	<50	8	3.87	3.63; 4.11	26.64 (*P* < 0.001)	8	3.54	3.20; 3.89	22.33 (*P* < 0.001)	8	4.27	3.80; 4.74	1.53 (*P* = 0.22)	8	3.73	3.46; 4.00	33.29 (*P* < 0.001)
	>50	9	4.72	4.50; 4.95		9	4.68	4.36; 5.01		9	4.68	4.24; 5.13		9	4.81	4.56; 5.06	

*k* = number of studies analysed for each subscale; *M* = mean effect size (sample means); *Q*_B_ = between‐groups heterogeneity statistic.

The moderator analyses for continuous variables are summarised in Table 5. As can be observed, the age of the participants of the primary studies and the gender (in proportions) of these participants did not show statistically significant results in the meta‐regression analyses.

**Table 5:** Meta‐regression analysis for continuous variables for each subscale

ASQ Subscale	Age			Gender		
	*k*	*b* [95% CI]	*t*	*k*	*b* [95% CI]	*t*
Sexual Rights	17	−0.01 [−0.04; 0.01]	−1.10 (*P* = 0.29)	17	−0.49 [−2.46; 1.48]	−0.53 (*P* = 0.60)
Parenting	17	−0.03 [−0.07; 0.01]	−1.81 (*P* = 0.09)	17	−0.99 [−3.66; 1.68]	−.79 (*P* = 0.44)
Non‐reproductive Sexual Behaviour	17	−0.02 [−0–05; 0.02]	−0.95 (*P* = 0.35)	17	0.62 [−1.86; 3.10]	0.54 (*P* = 0.60)
Self‐Control	17	−0.01 [−0.05: 0.02]	−0.69 (*P* = 0.50)	17	−0.35 [−2.78; 2.09]	−0.30 (*P* = 0.77)

## Discussion

Current attitudes towards the sexuality of adults with ID play an important role in their experience of sexuality. Understanding current attitudes, and which variables explain differences between studies, is crucial to be able to design effective interventions that are adjusted to peoples' attitudes. Based on previous studies, this meta‐analysis expected that differences would emerge according to the country of origin of the study (especially according to the associated per capita income as an index of socio‐economic development and related level of individualism), sample type (family, staff, general population), age and gender. This was partially supported by the data (age and gender were not related to more favourable attitudes).

The comparison of the studies by continent of origin did not yield any statistically significant results. However, this could be expected, as differences were proposed according to cultural orientation variables (Ditchman *et al*. 2017) attributed to the country of study, or socio‐economic indicators of development (Lottes and Alkula 2011) according to the annual GDP per capita data used for this study. Both variables yielded significant results for sexual rights, parenting and self‐control.

Levels of individualism or collectivism refer, in general terms, to the degree in which individual interests prevail over group interests, or vice versa (Hofstede *et al*. 2010). Higher levels of individualism could be related to a relaxation of norms surrounding the topic of marriage and sexuality (Twenge *et al*. 2015), probably due to less pressure to ‘fit in’ (Rahman *et al*. 2020) and a focus on pleasure and personal goals (Triandis 1995; Lo *et al*. 2010). In more collectivistic cultures, opinions and ideas are more determined by the group membership and the state ideology (Hofstede *et al*. 2010). Therefore, positions towards sexuality are more linked to societal thinking. Indeed, the included studies from countries with lower attributed individualism scores describe their outcomes as linked to their countries' norms and values, including religious influences (Chou *et al*. 2018; Winarni *et al*. 2018). Religion has previously been related to less favourable attitudes towards general sexuality (Lefkowitz *et al*. 2004; Lottes and Alkula 2011).

The socio‐economic development of a country measured by GDP has also been related to sexuality issues (Lottes and Alkula 2011). More specifically, this meta‐analysis shows that samples from the higher socio‐economic group hold more favourable attitudes towards the sexuality of adults with ID. The socio‐economic development of a country has been related to changes in ideological aspects, including more tolerant sexual norms (Inglehart and Welzel 2005; Lottes and Alkula 2011) and more egalitarian gender attitudes (Lippa *et al*. 2010). In general, a country's socio‐economic development is accompanied by other social and political circumstances that may affect the societal thinking and thus explain the observed and past relationships with sexuality issues.

Per capita income data is also related to a country's attributed level of individualism or collectivism (Hofstede *et al*. 2010). Therefore, the level of individualism and the socio‐economic development of a country may explain attitudinal differences jointly, as correlated factors, rather than independently. The combination of different cultural variables (i.e. income, religious background and gender empowerment) could be behind different patterns in attitudes towards different sexuality issues (Lottes and Alkula 2011), so this could also be expected to explain cross‐countries differences in attitudes towards the sexuality of adults with ID. These findings may be particularly relevant, as even if individual differences exist, they highlight that context and general thinking, or the way in which many different aspects of life are socially conceived, are crucial to attitudes and in turn the support given.

According to our results, general population and sector workers held more favourable attitudes than family members for the sexual rights and parenting subscales, as expected (Aunos and Feldman 2002;Lam *et al*. 2021; Correa *et al*. 2022). However, the fact that the general population hold the most favourable attitudes may be related to an important aspect. Participants may have reported their attitudes guided by a sense of political correctness, as this can be perceived as a sensitive topic. In addition, this population is expected to have little knowledge about the general functioning of adults with intellectual disabilities, which may have interfered with the adjustment of their responses. The lack of knowledge coupled with the need to give a politically correct answer may be denoting discomfort in answering these questions. This may suggest that the general population perceives a significant gap between the lifestyles, goals, needs and desires of adults with intellectual disabilities and adults without disabilities. Ultimately, this may reflect a problem involving a lack of acceptance of the adult with a disability as any other adult, regardless of their support needs.

Most of the studies that included family samples were composed of parents. Parents may hold more overprotective attitudes and behaviours, due to the cultural aspects by which they have learned about sexuality (Evans *et al*. 2009). Parents of adults with ID are usually older, and age could be an explanatory factor for these differences (Cuskelly and Bryde 2004). Also, misconceptions about sexuality and disability, linked to the reported lack of information and education on the issue (Evans *et al*. 2009), may also explain the observed differences. Interestingly, parents of people with ID are more supportive of the sexuality of their own child with ID than of others with ID (Karellou 2003). Therefore, the degree of perceived responsibility over the person with ID and involvement in their life plan could be an additional factor to consider when addressing this issue.

According to our results, age did not explain differences in attitudes between studies. This result was unexpected, as it seemed to be widely accepted in systematic review studies (Lam *et al*. 2021; Correa *et al*. 2022; Pebdani and Tashjian 2022). However, this could be explained by the characteristics of the included samples. According to the current literature, older samples hold less favourable attitudes compared with younger ones (Oliver *et al*. 2002; Meaney‐Tavares and Gavidia‐Payne 2012). For this analysis, a mean age value was used to compare studies. The youngest sample had a mean age value of 20 and the oldest had one of 54, but the majority was in the 30–45 age range. Therefore, comparisons between the oldest and the youngest were not representative enough. The expected relationship with age seemed logical, as age would reflect the societal thinking of each generation, and current ideologies towards sexuality and relationships seem to be more favourable and diverse than decades ago. This has already been found for sexual permissiveness regarding premarital and extramarital sexuality (Thornton and Young‐DeMarco 2001; Kraaykamp 2002) and non‐marital co‐habitation (Thornton and Young‐DeMarco 2001).

Finally, gender was not related to attitudes in our results. Although we expected there to be a relationship between gender and attitudes, it is true that previous literature has already cautioned that this relationship is unclear (Lam *et al*. 2021; Correa *et al*. 2022). However, when found, researchers have proposed that women may identify themselves as historically sexually repressed and therefore are more likely to promote the sexual rights of others (Ditchman *et al*. 2017). A meta‐analytic review on general attitudes and sexual behaviour examining gender differences proposed that gender differences in sexuality and attitudes are in fact smaller than researchers think, and when they exist, they may be explained by biological factors, societal power differentials and social pressures to assigned roles (Petersen and Hyde 2011), which could explain the lack of relatedness.

Limitations should be addressed. Relevant variables related to attitudes may have been missed. The variables included in this meta‐analysis were based on previous literature and the possibilities offered by the data available in the included studies. However, it is possible that other variables may underlie attitudinal differences. In terms of the interpretability of the results and as highlighted previously, the representativeness of the age variable was compromised by a narrow age range, limited to availability in the included studies. In addition, studies involving survey designs are susceptible to particular biases. In this case, especially respondents from the general population may have been guided by a sense of political correctness, as they have little knowledge about disability. Only studies using the ASQ‐ID measure were included. Therefore, relevant information provided by studies with other measures may have been missed in order to preserve maximum comparability of the attitudinal measure.

In addition, other methodological limitations should be mentioned. Due to the nature of the studies analysed, risk of bias analyses were not conducted in the present meta‐analysis. The studies included in this meta‐analysis do not present the same risks in the design, conduct, analyses and reporting as other kind of studies, as, for example, revisions including randomised trials, interventional or observational studies (e.g. Higgins and Green 2011). Therefore, no differences would be detected for the risk of bias measures in this sample of studies. However, Egger's and Kendall's tests for publication bias were included in the analyses, showing that there is not any tendency of publication bias in the sample of studies analysed in the present meta‐analysis.

Despite these limitations, we consider that the present study yields relevant information to the field and more specifically to the field of attitudinal studies on the issue. First, it is because to our knowledge, it is the first meta‐analytical approach to this issue, comparing studies across different contexts and countries. Second, it is because it allows to reinforce the known relationship between attitudes and aspects related to the country context, especially socio‐economic and cultural orientation (measured by individualism and collectivism) factors. Third, it is because it explores the relationship with age and gender in greater depth.

Due to the nature of the analysed studies, the sample mean was selected as the effect size measure for the present meta‐analysis, as so far there are no available primary studies in the existing literature testing comparisons between different groups in their ASQ‐ID responses. Future studies should deeply analyse potential sources of differences between groups of interest. For this purpose, we think that the moderator variables analysed in this study can be useful to design future research. This study provides important information for professionals and families when approaching the topic of sexuality of adults with ID, who should consider how attitudes may be limiting their interventions and support actions and be aware of how different populations may react to the sexuality of adults with ID. Particular perceptions should not limit desires and access to a healthy sexual life for anyone, regardless of a person's disability. On the other hand, adequate support must be provided. Likewise, for professionals involved in health promotion and sexual education, we suggest that they include information towards the normalisation of sexuality among people with disabilities in their interventions and actions. This could be crucial in bringing about an attitudinal change in the population. If we intend to help those adults with ID who want to live their sexuality more efficiently, it is crucial to understand to what extent they are experiencing barriers so that we can direct the necessary efforts to help them.

Finally, these intervention and practical proposals should be considered without ignoring an important and consistent finding across this meta‐analysis: the significant differences between countries. In this sense, any proposal may seem utopian, given that these attitudinal differences may be marked by societal thinking, religion and how general sexuality and romantic relationships are perceived. Furthermore, perhaps the way disability is viewed in these areas or how having a family member with a disability is experienced could have an important impact. Therefore, a double reading can be made: First, intervention becomes even more urgent than in other geographical areas, as we should not forget that we are talking about a right, which in those cases is somewhat denied for adults with intellectual disabilities. And second, any action should emphasise some general aspects, such as general human sexuality and functioning, interpersonal relationships, human rights and the rights of adults with intellectual disabilities as analogous and identical to those of any other person. Of course, all of this should be done while maintaining a balance with the utmost respect for all cultures and ideologies.

## Conclusions

People's attitudes towards the sexuality of adults with ID are a major barrier to adults with ID who want to experience their sexuality (Healy *et al*. 2009). Several variables have been related to more or less favourable attitudes, this study being the first meta‐analytic approach to the topic.

Belonging to the general population or being a staff member, being from more individualistic countries and belonging to countries with a higher socio‐economic development are related to more favourable attitudes. By contrast, we did not find the expected relationship with gender or age. The latter one could be explained by methodological issues, as differences would be expected when comparing younger and older participants, according to previous studies (Oliver *et al*. 2002; Meaney‐Tavares and Gavidia‐Payne 2012).

Therefore, this study leads us to propose that factors regarding the societal and ideological thinking of the population may be limiting attitudes towards the sexuality of adults with ID. According to these findings, factors related to the context in which we grow up may be determining our way of thinking about an issue that should be seen for what it is—a right. This is extremely relevant. Therefore, raising awareness and training actions should be carried out considering the general and specific ideological background of the target populations, in order to support people with disabilities in fulfilling their sexual rights in an effective and healthy way.

## Source of funding

This work was supported by the Government of Aragon (Group S31_20D), Department of Innovation, Research and University and FEDER 2020‐2022, ‘Building Europe from Aragón’.

## Conflict of interest

All authors declare they have no conflict of interest.

## Supporting information


**Data S1.** Supporting Information

## Data Availability

Data sharing is not applicable to this article as no new data were created or analysed in this study.

## References

[jir12971-bib-0001] Aunos M. & Feldman M. A. (2002) Attitudes towards sexuality, sterilization and parenting rights of persons with intellectual disabilities. Journal of Applied Research in Intellectual Disabilities 15, 285–296.

[jir12971-bib-0002] Bergmüller S. (2013) The relationship between cultural individualism‐collectivism and student aggression across 62 countries: student aggression and culture. Aggressive Behavior 39, 182–200. 23494751 10.1002/ab.21472

[jir12971-bib-0003] Borenstein M. , Hedges L. V. , Higgins J. P. T. & Rothstein H. R. (2010) A basic introduction to fixed‐effect and random‐effects models for meta‐analysis. Research Synthesis Methods 1, 97–111. 26061376 10.1002/jrsm.12

[jir12971-bib-0004] Botella J. & Sánchez‐Meca J. (2015) Meta‐análisis en ciencias sociales y de la salud. [Meta‐analysis in social sciences and health]. Editorial Síntesis.

[jir12971-bib-0005] Central Intelligence Agency . (n.d.) The world factbook: GDP—per capita (PPP). Available at: https://www.cia.gov/the‐world‐factbook/field/real‐gdp‐per‐capita/ (retrieved 29 july 2021).

[jir12971-bib-0006] Chou Y.‐C. , Lu Z. J. & Lin C.‐J. (2018) Comparison of attitudes to the sexual health of men and women with intellectual disability among parents, professionals, and university students. Journal of Intellectual & Developmental Disability 43, 164–173.

[jir12971-bib-0007] Correa A. B. , Castro Á. & Barrada J. R. (2022) Attitudes towards the sexuality of adults with intellectual disabilities: a systematic review. Sexuality and Disability 40, 261–297.

[jir12971-bib-0008] Cuskelly M. & Bryde R. (2004) Attitudes towards the sexuality of adults with an intellectual disability: parents, support staff, and a community sample. Journal of Intellectual & Developmental Disability 29, 255–264.

[jir12971-bib-0009] Cuskelly M. & Gilmore L. (2007) Attitudes to sexuality questionnaire (individuals with an intellectual disability): scale development and community norms. Journal of Intellectual & Developmental Disability 32, 214–221. 17885900 10.1080/13668250701549450

[jir12971-bib-0010] Deffew A. , Coughlan B. , Burke T. & Rogers E. (2022) Staff member's views and attitudes to supporting people with an intellectual disability: a multi‐method investigation of intimate relationships and sexuality. Journal of Applied Research in Intellectual Disabilities 35, 1049–1058. 34009719 10.1111/jar.12897

[jir12971-bib-0011] Di Marco G. , Licciardello O. , Mauceri M. & La Guidara R. M. C. (2013) Attitudes towards the sexuality of men with intellectual disability: the effect of social dominance orientation. Procedia ‐ Social and Behavioral Sciences 84, 1194–1198.

[jir12971-bib-0012] Ditchman N. , Easton A. B. , Batchos E. , Rafajko S. & Shah N. (2017) The impact of culture on attitudes toward the sexuality of people with intellectual disabilities. Sexuality and Disability 35, 245–260.

[jir12971-bib-0013] Esterle M. , Munoz Sastre M. T. & Mullet E. (2008) Judging the acceptability of sexual intercourse among people with learning disabilities: French laypeople's viewpoint. Sexuality and Disability 26, 219–227.

[jir12971-bib-0014] Evans D. S. , McGuire B. E. , Healy E. & Carley S. N. (2009) Sexuality and personal relationships for people with an intellectual disability. Part II: staff and family carer perspectives. Journal of Intellectual Disability Research 53, 913–921. 19761470 10.1111/j.1365-2788.2009.01202.x

[jir12971-bib-0015] Franco D. G. , Cardoso J. & Neto I. (2012) Attitudes towards affectivity and sexuality of people with intellectual disability. Sexuality and Disability 30, 261–287.

[jir12971-bib-0016] Gilmore L. & Chambers B. (2010) Intellectual disability and sexuality: attitudes of disability support staff and leisure industry employees. Journal of Intellectual & Developmental Disability 35, 22–28. 20121663 10.3109/13668250903496344

[jir12971-bib-0017] Grieve A. , McLaren S. , Lindsay W. & Culling E. (2009) Staff attitudes towards the sexuality of people with learning disabilities: a comparison of different professional groups and residential facilities. British Journal of Learning Disabilities 37, 76–84.

[jir12971-bib-0018] Healy E. , McGuire B. E. , Evans D. S. & Carley S. N. (2009) Sexuality and personal relationships for people with an intellectual disability. Part I: service‐user perspectives. Journal of Intellectual Disability Research 53, 905–912. 19709348 10.1111/j.1365-2788.2009.01203.x

[jir12971-bib-0019] Hedges L. V. & Vevea J. L. (1998) Fixed‐ and random‐effects models in meta‐analysis. Psychological Methods 3, 486–504.

[jir12971-bib-0020] Higgins J. & Green S. (eds) (2011) Cochrane handbook for systematic reviews of interventions version 5.1.0 [updated March 2011]. The Cochrane Collaboration Available at: www.handbook.cochrane.org (retrieved 1 june 2011).

[jir12971-bib-0021] Hofstede G. , Hofstede G. J. & Minkov M. (2010) Cultures and organizations. Software of the mind, 3rd edn. McGraw‐Hill, New York.

[jir12971-bib-0022] Huedo‐Medina T. B. , Sánchez‐Meca J. , Marín‐Martínez F. & Botella J. (2006) Assessing heterogeneity in meta‐analysis: Q statistic or I^2^ index? Psychological Methods 11, 193–206. 16784338 10.1037/1082-989X.11.2.193

[jir12971-bib-0023] Inglehart R. & Welzel C. (2005) Modernization, cultural change, and democracy, the human development sequence. Cambridge University Press, New York.

[jir12971-bib-0024] IntHout J. , Ioannidis J. P. & Borm G. F. (2014) The Hartung‐Knapp‐Sidik‐Jonkman method for random effects meta‐analysis is straightforward and considerably outperforms the standard DerSimonian‐Laird method. BMC Medical Research Methodology 14, 1–12. 24548571 10.1186/1471-2288-14-25 PMC4015721

[jir12971-bib-0025] Karellou J. (2003) Laypeople's attitudes towards the sexuality of people with learning disabilities in Greece. Sexuality and Disability 21, 65–84.

[jir12971-bib-0026] Kraaykamp G. (2002) Trends and countertrends in sexual permissiveness: three decades of attitude change in the Netherlands 1965‐1995. Journal of Marriage and Family 64, 225–239.

[jir12971-bib-0027] Lam A. , Yau M. , Franklin R. & Leggat P. A. (2021) Public opinion on the sexuality of people with intellectual disabilities: a review of the literature. Sexuality and Disability 39, 395–419.

[jir12971-bib-0028] Lefkowitz E. S. , Gillen M. M. , Shearer C. L. & Boone T. L. (2004) Religiosity, sexual behaviors, and sexual attitudes during emerging adulthood. The Journal of Sex Research. 41, 150–159. 15326540 10.1080/00224490409552223

[jir12971-bib-0029] Lippa R. A. , Collaer M. L. & Peters M. (2010) Sex differences in mental rotation and line angle judgments are positively associated with gender equality and economic development across 53 nations. Archives of Sexual Behavior 39, 990–997. 19130205 10.1007/s10508-008-9460-8

[jir12971-bib-0030] Lipsey M. W. & Wilson D. B. (2001) Practical meta‐analysis. Sage. Available at: https://us.sagepub.com/en‐us/nam/practical‐meta‐analysis/book11092

[jir12971-bib-0031] Lo V. , So C. Y. K. & Zhang G. (2010) The influence of individualism and collectivism on Internet pornography exposure, sexual attitudes, and sexual behavior among college students. Chinese Journal of Communication. 3, 10–27.

[jir12971-bib-0032] Lottes I. L. & Alkula T. (2011) An investigation of sexuality‐related attitudinal patterns and characteristics related to those patterns for 32 European countries. Sexuality Research & Social Policy 8, 77–92.

[jir12971-bib-0033] Meaney‐Tavares R. & Gavidia‐Payne S. (2012) Staff characteristics and attitudes towards the sexuality of people with intellectual disability. Journal of Intellectual & Developmental Disability 37, 269–273. 22838968 10.3109/13668250.2012.701005

[jir12971-bib-0034] Moher D. , Shamseer L. , Clarke M. , Ghersi D. , Liberati A. , Petticrew M. *et al*. (2015) Preferred reporting items for systematic review and meta‐analysis protocols (PRISMA‐P) 2015 statement. Systematic Reviews 4, 1–9. 25554246 10.1186/2046-4053-4-1 PMC4320440

[jir12971-bib-0035] Morales G. E. M. , Ramirez E. O. L. , Esterle M. , Sastre M. T. M. & Mullet E. (2010) Judging the acceptability of sexual intercourse among people with learning disabilities: a Mexico‐France comparison. Sexuality and Disability 28, 81–91.

[jir12971-bib-0036] Oliver M. N. , Anthony A. , Leimkuhl T. T. & Skillman G. D. (2002) Attitudes toward acceptable socio‐sexual behaviors for persons with mental retardation: implications for normalization and community integration. Education and Training in Mental Retardation and Developmental Disabilities 37, 193–201.

[jir12971-bib-0037] Pebdani R. N. (2016) Attitudes of group home employees towards the sexuality of individuals with intellectual disabilities. Sexuality and Disability 34, 329–339.

[jir12971-bib-0038] Pebdani R. N. & Tashjian A. (2022) An analysis of the attitudes of the general public towards the sexuality of individuals with disabilities through a systematic literature review. Sexuality and Disability 40, 21–55.

[jir12971-bib-0039] Petersen J. L. & Hyde J. S. (2011) Gender differences in sexual attitudes and behaviors: a review of meta‐analytic results and large datasets. The Journal of Sex Research. 48, 149–165. 21409712 10.1080/00224499.2011.551851

[jir12971-bib-0040] R Development Core Team (2021) R: a language and environment for statistical computing. Vienna, Austria, R Foundation for Statistical Computing. ISBN 3‐900051‐07‐0. Available at: http://www.R‐project.org/

[jir12971-bib-0041] Rahman Q. , Xu Y. , Lippa R. A. & Vasey P. L. (2020) Prevalence of sexual orientation across 28 nations and its association with gender equality, economic development, and individualism. Archives of Sexual Behavior 49, 595–606. 31797225 10.1007/s10508-019-01590-0 PMC7031179

[jir12971-bib-0042] Raundenbush S. W. (2009) Analyzing effect sizes: Random‐effects models. In: The handbook of research synthesis and meta‐analysis (eds H. Cooper , L. V. Hedges & J. C. Valentine ), 2nd edn, pp. 295–315. Russell Sage Foundation.

[jir12971-bib-0043] Ryan D. & McConkey R. (2000) Staff attitudes to sexuality and people with intellectual disabilities. The Irish Journal of Psychology. 21, 88–97.

[jir12971-bib-0044] Sankhla D. & Theodore K. (2015) British attitudes towards sexuality in men and women with intellectual disabilities: a comparison between white westerners and South Asians. Sexuality and Disability 33, 429–445. 26594077 10.1007/s11195-015-9423-7 PMC4643113

[jir12971-bib-0045] Schwarzer G. (2007) meta: An R package for meta‐analysis. R News 7, 40–45.

[jir12971-bib-0046] Sinclair J. , Unruh D. , Lindstrom L. & Scanlon D. (2015) Barriers to sexuality for individuals with intellectual and developmental disabilities: a literature review. Education and Training in Autism and Developmental Disabilities. 50, 3–16.

[jir12971-bib-0047] Stoffelen J. M. T. , Herps M. A. , Buntinx W. H. E. , Schaafsma D. , Kok G. & Curfs L. M. G. (2017) Sexuality and individual support plans for people with intellectual disabilities: a study on the content of ISP. Journal of Intellectual Disability Research 61, 1117–1129. 29024162 10.1111/jir.12428

[jir12971-bib-0048] Tamas D. , Brkic Jovanovic N. , Rajic M. , Bugarski Ignjatovic V. & Peric Prkosovacki B. (2019) Professionals, parents and the general public: attitudes towards the sexuality of Persons with Intellectual Disability. Sexuality and Disability 37, 245–258.

[jir12971-bib-0049] Thornton A. & Young‐DeMarco L. (2001) Four decades of trends in attitudes toward family issues in the United States: the 1960s through the 1990s. Journal of Marriage and Family 63, 1009–1037.

[jir12971-bib-0050] Triandis H. C. (1995) Individualism and collectivism. Westview Press, Boulder, CO.

[jir12971-bib-0051] Twenge J. M. , Sherman R. A. & Wells B. E. (2015) Changes in American adults' sexual behavior and attitudes, 1972–2012. Archives of Sexual Behavior 44, 2273–2285. 25940736 10.1007/s10508-015-0540-2

[jir12971-bib-0052] Ubillos S. , Paez D. & González J. L. (2000) Culture and sexual behavior. Psicothema 12, 70–82.

[jir12971-bib-0053] Viechtbauer W. (2010a) Conducting meta‐analyses in *R* with the metafor package. Journal of Statistical Software 36, 1–48.

[jir12971-bib-0054] Viechtbauer, W. (2010b) Metafor: meta‐analysis package for R. R package version 1.4‐0. Available at: http://CRAN.R‐project.org/package=metafor (retrieved 1 june 2011).

[jir12971-bib-0055] Winarni T. I. , Hardian H. , Suharta S. & Ediati A. (2018) Attitudes towards sexuality in males and females with intellectual disabilities: Indonesia setting. Journal of Intellectual Disability ‐ Diagnosis and Treatment. 6, 43–48.

